# Correction to ‘Circulating miRNA in functional tricuspid regurgitation. Unveiling novel links to heart failure: A pilot study’

**DOI:** 10.1002/ehf2.70018

**Published:** 2025-11-16

Hinojar, R. , Moreno‐Gómez‐Toledano, R. , Conde, E. , Gonzalez‐Gomez, A. , García‐Martin, A. , González‐Portilla, P. , Fernández‐Golfín, C. , García‐Bermejo, M. L. , Zaragoza, C. , and Zamorano, J. L. (2024) Circulating miRNA in functional tricuspid regurgitation. Unveiling novel links to heart failure: A pilot study. ESC Heart Failure, 11: 2272–2286. 10.1002/ehf2.14765 38638083 PMC11287356.

In the Funding section, the current text states: *“This study was supported by the Instituto de Salud Carlos III, PI20/01206.”*

However, it should read: *“This study was supported by the Instituto de Salud Carlos III, PI20/01206, and co‐funded by the European Union.”*

We apologize for this error.

